# Opinions, training and requirements regarding ICT of educators in Florence and Granada for students with functional diversity

**DOI:** 10.1007/s10209-023-00977-0

**Published:** 2023-02-23

**Authors:** Carmen del Pilar Gallardo-Montes, María Jesús Caurcel Cara, Antonio Rodríguez Fuentes, Davide Capperucci

**Affiliations:** 1 Department of Didactics and School Organization at the Faculty of Education, Faculty of Education, University of Granada, Campus “La Cartuja” S/N, 18071 Granada, Spain; 2 Department of Developmental and Educational Psychology, Faculty of Education, University of Granada, Campus “La Cartuja” S/N, 18071 Granada, Spain; 3 Department of Training, Languages, Interculture, Literature and Psychology, University of Florence, Street Laura, 48, 50121 Florence, Italy

**Keywords:** Special needs education, Improving classroom teaching, Teacher professional development, Educational technology, Teacher training

## Abstract

Information and communication technology (ICT) is a means of learning for people with functional diversity (FD), in the context of both formal and non-formal education. These digital options favor their inclusion, participation and motivation, so having competent professionals in the field of educational technology is crucial for the full development of this population. Within this framework, the opinion, training and requirements regarding ICT of 809 educators in Florence (Italy) and Granada (Spain) were analyzed, following a quantitative study, with a non-experimental, descriptive, cross-sectional and comparative design. Descriptive statistics (mean, standard deviation and mode) and frequencies were used. After checking the normality of the data (Kolmogórov–Smirnov test), inferential analyses were performed, applying Student's *t*- test and one-factor ANOVA, calculating the effect size (Cohen’s *d* and eta squared). Statistically significant differences were found according to city of origin, gender, and years of experience with students with FD. The results point to the need for different educators to have sufficient technological training, as despite being aware of the functionalities of ICT and having a favorable opinion of them, resources and knowledge about their use fall short of what is desirable.

## Introduction

Information and communication technologies (ICTs) are central to professional work in any sector, and have become essential in modern society. Nevertheless, the social reality—where ICTs are key players in the interaction—is some way ahead of educational reality [1]. Despite their rapid growth and the transformations they have brought about in family, cultural, political, social and school environments [2], there is continued reticence over their incorporation into the educational panorama in terms of teaching practice, and they have only been introduced to this context belatedly [3].

ICT facilitates access to the educational world [4], and improves the care and quality of teaching for student diversity. Therefore, directing teaching toward practices that include technology is crucial to achieving meaningful learning for the totality of students, regardless of their capabilities and/or difficulties [5]. Likewise, orienting the current educational model toward the inclusion of ICT in the classroom entails understanding education in a broader and more attractive way for students [2].

Thus, there is demand for the educational system to be updated [6], from early up to higher educational stages, not only in terms of the inclusion of ICTs in classrooms, but also regarding the teaching of them. Technology should not be seen as an isolated aspect but as a means to achieving educational ends [7].

The impact of ICTs for students is enriching and facilitates learning [5], opening up a world of possibilities to students with functional diversity (FD) [4, 8, 9] and improving the quality of education, teacher performance [4, 10, 11] and educational inclusion [12]. Just as ICT forms part of teaching practice in ordinary classrooms, students with FD form part of those classrooms and also benefit from the potential ICTs offer, since they promote communication [13–16], participation [17], integration [18] and equality of opportunity [4, 14]. Given the interest ICTs arouse in students of diversity, and the benefits they can provide in the capacities and aptitudes these students can develop, tools and materials in digital format designed specifically for them have proliferated. Rodríguez [19] examines alternative access to such media for people with sensory disorders. Gutiérrez and Martorell [20] study the behavior patterns of people with mental disabilities when using ICT. Zubillaga and Alba [21] analyze the possibilities of ICT regarding access and participation in the educational curriculum of students with FD. Moreover, given the explosion of smartphone apps, some authors, such as Abdul et al. [22], Gallardo-Montes et al. [23], Hajjar et al. [24] and Wang and Hsu [25], investigate and implement the use of apps designed for people with ADHD, ASD or Down’s syndrome.

It is not enough for teachers to know and handle technologies in the classroom if they do not know how to utilize and apply them [26]. Teachers should be suitably trained with the necessary knowledge on the difficulties that technologies cause for students and overcome barriers in learning [17].

Therefore, digital competence is key to consolidating these tools in a pedagogical manner in classrooms [7]. Their relevance is made clear in the educational legislation of various countries, including Italy and Spain [27, 28], pushing both teachers and students to be competent in the digital sphere. The development and implementation of this competence in schools requires specific effort from teachers [4], and school management teams, who play a vital role in the integration of ICT in the classroom, alongside education administrations and the students themselves [29].

In spite of the benefits and possibilities that ICT offers for developing many skills in students with FD, many teachers still have not developed a suitable attitude with regard to technology and their handling of diversity, whether due to lack of training, lack of knowledge, and/or the effort required to change from a more traditional methodology to a more interactive one based on digital resources.

A good number of authors have looked at the inclusion of ICT in diverse classrooms. García-Valcárcel et al. [30] show that, in the use of ICTs for collaborative learning, teachers value them as tools that motivate and integrate, that offer autonomy, grab student attention and adapt to their needs, but present difficulties in their use. Cabero-Almenara et al. [13] and Fernández-Batanero et al. [4] find limited training in applied technologies for attending to diversity among future primary teachers, and a lack of awareness of their benefits and functionality. Randazzo and Oteri [31] find positive attitudes held by university teachers toward ICT, but these teachers neither use nor have a good command of them. While the authors do not observe differences by sex, they do find them with teaching experience, with the longest-serving teachers being those who considered ICT to be an effective tool. Messina et al. [32] compare the use of ICT by Spanish and Italian teachers. Italian teachers have a low digital competence due to a lack of training and experience, and mainly use the computer and interactive whiteboard, followed by the smartphone and tablet. Spanish teachers use the computer often and occasionally the interactive whiteboard or tablet, and their digital competence is middling.

In the last few years there has been constant interest in the use of ICT in the educational field [33], which has only increased with the COVID-19 pandemic. Bunting et al. [34] observe that primary teachers include a range of digital tools to work in support of textbooks, due to their high quality. They show how teachers have become persuaded to implement a teaching model using ICT that is more personalized to their students. Nikolopoulou et al. [35] show that teachers with less experience are more disposed to apply mobile learning in the classroom. Sailer et al. [36] find that teachers’ digital skills have an influence on the integration of ICT as well as the training received. Yakubova et al. [37] reveal that the limited frequency of use of virtual and augmented reality with students with disabilities is connected to lack of training or technical support, and that younger teachers use it more. Yilmaz [38] states that the inclusion of different technologies foments the critical and creative thinking of future teachers, as well as academic achievements. Ardiç [39] concludes that teachers have positive attitudes toward ICTs, which significantly influences their application in the classroom.

With this in mind, and with the problem posed by the lack of generalization of didactic knowledge and daily use of ICT in the classrooms of various contexts in spite of their advantages, the aims of this study are:

1. To learn the opinion of educators from Florence and Granada about ICTs and their benefits for students with FD;
2. To discover the requirements and needs of using ICT in order to teach people with FD in the two countries;
3. To examine the training and experience that educators from Italy and Spain have with ICT for students with FD;
4. To find out which ICTs the educators from both countries use with students with FD;
5. To determine the opinion, training, and demands regarding ICT of the educators according to their city of origin, sex, and years of experience working with students with FD.

## Method

The study has adopted a quantitative approach with a non-experimental [40, 41], cross-sectional and descriptive-comparative design [42, 43].

### Participants

The study sample comprised 809 professionals from the education sector, coming from the cities of Florence (*F*), Italy (*n* = 488), and Granada (*G*), Spain (*n* = 321). Initially, there were 839 participants, of whom 30 were removed because they lacked the required pedagogical experience (recent graduates or people unconnected to teaching in formal or non-formal contexts). Non-probability convenience sampling was employed [44, 45, 46], [47, 48].

The participants from Granada consisted of 253 women (78.8%), 67 men (20.9%) and 1 “other” (0.3%), of whom 251 self-identified as female (78.2%) and 70 as male (21.8%) in gender. Bearing in mind the significant number of female participants, the sex and/or gender did not result in bias in this investigation, as studies in Social Science and Legal Science have a predominance of women [49, 50]. Ages ranged from 20 to 64 years old (*M* = 39.74, SD = 11.19). Most were non-specialist (35.2%), specialist (23.7%) and Special Needs (21.5%) teachers. From Florence, there were 449 women (92%), 38 men (7.8%) and 1 “other” (0.2%), of whom 450 (92%) self-identified as female, 37 (7.6%) as male, and 1 “other” (0.2%). The age range varied between 24 and 60 years old (*M* = 37.97, SD = 7.75). They mainly worked as non-specialist (48.8%) and support (65%) teachers. Table 1 shows other sociodemographic data and data on ICT of the sample.

**Table 1:** Other sociodemographic and ICT data of the participants

Variables		Florence *N* (%)	Granada *N* (%)
Experience with students with FD	No years	24 (4.9)	25 (7.8)
	≤ 5 years	236 (48.4)	146 (45.5)
	6–10 years	188 (38.5)	47 (14.6)
	11–20 years	29 (5.9)	61 (19)
	≥ 21 years	11 (2.3)	42 (13.1)
Diversity worked with	Oral language	131 (26.8)	163 (50.8)
	Written language	309 (63.3)	164 (51.1)
	Eating disorder	39 (8%)	17 (5.3)
	Mental disorder	61 (12.5)	34 (10.6)
	Behavioral	281 (57.6)	217 (67.6)
	Emotional	109 (22.3)	65 (20.2)
	Development	290 (59.4)	189 (58.9)
	Sensorial	109 (22.3)	84 (26.2)
	Intellectual	152 (31.1)	152 (47.4)
	Physical/Motor	157 (32.2)	77 (24)
Availability of internet access	Yes	462 (94.7)	317 (98.8)
	No	26 (5.3)	4 (1.2)
Type of ICT available in the school	None	15 (3.1)	4 (1.2)
	Tablet	211 (43.2)	153 (47.7)
	Smartphone	86 (17.6)	100 (31.2)
	Computer	435 (89.1)	301 (93.8)
	Projector	288 (59%)	217 (67.6)
	TV	85 (17.4)	75 (23.4)
	Interactive digital whiteboard	124 (25.4)	24 (7.5)

These were drawn up as multiple-response items (except “Experience with FD”)

### Instrument

In order to discover the opinion of the educators and the training on ICT for working with people with FD, we administered the Italian version of the questionnaire—“Questionario sulla formazione e sulle competenze legate all’uso delle TIC degli insegnati che operano con alunni disabili” [“Questionnaire on training and competences related to the use of ICT for teachers who work with disabled pupils”] [51], and the Spanish version, “Demandas y potencialidades de las TIC y las apps para la atención a personas con autismo (DPTIC-AUT-Q)” [“demands and potentials of ICT and Apps for assisting people with autism”] [ 52]. It had a section on sociodemographic data and four subscales connected to ICT: subscale 1: opinion, training and uses of ICT by professionals for teaching people with functional diversity; subscale 2: training and uses of ICT by professionals for teaching people with autism; subscale 3: uses and benefits of apps in assisting people with autism; subscale 4: uses and possibilities of apps specialized for people with autism.

In order to meet the aims of this study, we have only used the first subscale, “Opinion, training and uses of ICT by professionals for teaching people with functional diversity”, which comprised questions with Likert-scale responses (1 = completely disagree; 5 = completely agree). It had a multiple-answer section aimed at finding out the types of ICT the professionals used with people with FD, and had three dimensions: D1: opinion on ICTs for people with FD (items 1–11); D2: requirements and possibilities of ICT for teaching people with FD (items 12–16); D3: ICT training of professionals for assisting people with FD (items 17–22).

The questionnaire has adequate psychometric properties. It obtained excellent intraclass correlation coefficients in subscale 1 (Italian version = 0.954; Spanish version = 0.986); significant Kendall’s *W* inter-rater concordance, *p* < 0.001 (Italian version = 0.192 clarity; 0.197 coherence; 0.202 relevance; and 0.218 objectivity; Spanish version = 0.153 clarity; 0.150 coherence; 0.200 relevance; and 0.211 objectivity); and an exceptional internal consistency: *α*_*I*taly_Block_1_ = 0.982; *α*_Spain_Block_1_ = 0.950.

The results of the CFA for Subscale 1 were equally favorable and acceptable [53, 54]: the chi-square value was statistically significant (*χ*^*2*^ = 1592.286, *p* = 0.0000). All other values indicated an adequate instrument fit: RMSEA (0.001) and WRMR (1.039), demonstrating the goodness of the model. Cronbach’s coefficient was high for each factor (*α*__D1_ = 0.95; *α*__D2_ = 0.75; *α*__D3_ = 0.91), as was composite reliability (CR__D1_ = 0.93; CR__D2_ = 0.66; CR__D3_ = 0.88).

### Procedure

The study received a favorable report from the Human Research Ethics Committee [2002/CEIH/2021] of the University of Granada (Spain). In Italy, the questionnaires were administered during teacher further-education sessions on diversity at the Università degli Studi di Firenze (University of Florence). The participants were given the details of the study aims in person, explaining the voluntary nature of their participation, the anonymity of their data and the exclusivity of their employment for the purpose of research. In Granada, they were contacted by telephone, email and in person with schools and associations that attended to people with FD, requesting collaboration and explaining the research aims. The invitation link to the questionnaire, designed with the *LimeSurvey* platform*,* was provided by email, along with the aforementioned conditions of employment, being voluntary and anonymous.

### Data analysis

The data were analyzed with the SPSS v.25.0 statistics packet. We calculated descriptive statistics (mean, mode and standard deviation) and frequencies. Once we had tested the normality of the data (Kolmogórov-Smirnov test), parametric inferential analyses were carried out. For the dichotomous variables “city” and “sex”, Student’s *t-* test was used and the effect size calculated (Cohen’s *d*). For the variable “years of experience with FD”, the ANOVA *F*-test was used and the subsequent Tukey’s HSD and Bonferroni tests, along with the homogeneous subsets test, calculating the effect size using eta squared (*η*^*2*^).

## Results

In Table 2, according to the mean and mode values, we can see that the opinion of the participants on ICT for people with FD and their implementation in their professional work was located between options 4 (“agree”) and 5 (“completely agree”).

**Table 2:** Opinion on and training in using ICT with students with FD (*N* = 809)

ITEM		*M*	SD	*M*_*o*_	%				
					1	2	3	4	5
1. Opinion	1. They improve the teacher’s…	4.13	0.87	4	1.5	2.6	15.8	41.7	38.4
	2. They require assistance in…	4.06	0.79	4	0.9	2.2	16.4	51.3	29.2
	3. They give greater flexibility…	4.31	0.73	5	0.5	1.2	9.5	44.1	44.6
	4. They make it possible to meet…	4.18	0.77	4	0.5	1.1	15.6	45.5	37.3
	5. They are easy to use…	3.86	0.86	4	0.5	5.1	26.3	44.1	24.0
	6. They facilitate inclusion	4.09	0.84	4	0.7	3.0	17.4	44.1	34.7
	7. They offer multiple opportunities	3.82	0.75	4	0.9	3.6	22.9	58.1	14.6
	8. They improve performance and…	3.76	0.85	4	1.7	3.6	30.4	45.9	18.4
	9. They increase motivation…	4.23	0.76	4	1.0	1.0	11.1	47.3	39.6
	10. They enable access to information	4.25	0.70	4	0.6	0.6	9.5	51.7	37.6
	11. They make it possible to achieve…	4.10	0.78	4	0.5	2.6	14.7	50.4	31.8
2. Requirements	12. They need greater dedication…	3.49	1.04	4	4.1	13.0	28.6	38.6	15.8
	13. They require specific training…	4.28	0.75	4	0.6	1.7	8.7	46.6	42.4
	14. They need greater material means	4.36	0.77	5	0.5	2.0	9.1	37.3	51.5
	15. They help pay more attention	4.17	0.83	4	1.1	2.1	14.7	42.9	39.2
	16. I know how to choose specific…	3.51	0.83	4	1.2	8.3	38.3	42.3	9.9
3. Training	17. I know the main limitations	3.55	0.83	4	1.9	6.8	35.6	45.7	10.0
	18. I know different websites of…	3.72	0.85	4	1.4	6.8	25.3	51.2	15.3
	19. I know how to design activities…	3.26	0.99	4	5.7	15	35.0	36.7	7.7
	20. I feel ready to help them…	3.52	0.88	4	2.1	10.1	31.0	47.1	9.6
	21. It provides me with designs and…	3.92	0.81	4	0.7	3.8	21.1	51.7	22.6
	22. They help carry out assessment	3.79	0.87	4	1.6	4.2	28.2	45.6	20.4

*M* = Mean; SD = Standard deviation; *M*_*o*_ = Mode

Requirements and possibilities

In dimension 1, concerning opinion about ICT for people with FD, the participants revealed that these digital options enabled access to information in a flexible way and increased student motivation, while meeting their educational needs and improving the teacher’s competences. However, they agreed less on their ease and opportunities for use, and on their improving student performance.

For dimension 2, regarding the requirements and possibilities of ICT, it was mainly shown that they help to pay better attention, but that their use in the classroom with people with FD involved greater investment by the administration and specific training for teachers.

Dimension 3, in respect to teacher training in ICT, produced means that were slightly lower that the other dimensions. It showed that the teachers knew to a lesser extent how to design activities with general educational software and felt less prepared to help students in the use of technical support and use of ICT.

Only 5.7% of the participants from Florence (F) and 4.4% of those from Granada (*G*) did not use ICT with their students with FD. The computer (77.3% *F*; 83.2% *G*) and the tablet (45.7% *F*; 49.2% *G*) were the options most used by teachers, followed by the projector (38.7% *F*; 51.0% *G*), the smartphone (30.3% *F*; 22.7% *G*), the interactive whiteboard (17.6% *F*; 5.9% *G*), and the television (5.1% *F*; 11.5% *G*).

As a function of “city”, there were statistically significant differences in most items (*p* < 0.05) (Table 3). In the dimension of opinion on ICT (items 1–11), the participants from Granada were different in items 1, 2, 3, 4, 8, 9, and 11, obtaining higher means than the Florentine teachers, with an effect size between small (*d* > 0.20) and medium (*d* > 0.50). These items referred to the improvement in competences the ICT afforded the teacher, the assistance required for their use, the flexibility and educational solution they gave and the increase in performance, and the efficacy and motivation that they produced in the students.

**Table 3:** Significant differences in the opinion, training and requirements regarding ICT as a function of the city

Item	Florence		Granada		*t*	*d*
	*M*	SD	*M*	SD		
1	3.93	0.90	4.43	0.74	− 8.24*	0.61
2	3.94	0.76	4.23	0.80	− 5.26*	0.37
3	4.25	0.74	4.40	0.71	− 2.95*	0.10
4	4.10	0.75	4.30	0.78	− 3.50*	0.26
8	3.58	0.85	4.03	0.78	− 7.68*	0.55
9	4.08	0.75	4.47	0.72	− 7.26*	0.53
10	4.13	0.67	4.43	0.70	− 6.18*	0.44
11	4.05	0.77	4.19	0.78	− 2.57*	0.18
12	3.25	1.01	3.85	0.97	− 8.42*	0.61
14	4.27	0.81	4.51	0.69	− 4.52*	0.32
15	4.00	0.84	4.42	0.76	− 7.16*	0.52
17	3.49	0.76	3.64	0.94	− 2.47*	0.18
18	3.63	0.79	3.87	0.93	− 3.99*	0.28
19	3.41	0.84	3.03	1.16	5.34*	0.38
20	3.61	0.79	3.39	0.99	3.55*	0.25
22	3.69	0.87	3.95	0.85	− 4.22*	0.30

*M* = Mean; SD = Standard deviation; *t* = Student’s *t*, *d* = Cohen’s *d*

*p < .05

In terms of the requirements and possibilities of ICT (items 12–16), there were statistically significant differences in three of the five items, in this case resulting in higher means for the participants from Granada in items 12, 14 and 15, which concerned dedication and material investment required by the ICT, and the help that these tools offered for looking after diversity.

In the dimension regarding didactic training for teachers in the use of ICT (items 17–22), the Florentine participants obtained higher means with a medium effect size in the questions concerning the preparation for using ICT to help students with the use of digital tools, and for the design of activities with general educational software. However, the participants from Granada scored significantly higher, and with a medium and small effect size, in the items regarding knowledge of the limitations the students might have in using ICT, the finding of specific resources on the internet, and the simplicity that technologies provided when it came to carrying out assessment.

Given that the independent variable referring to location determined significant differences, the rest of the contrasts were carried out with the two samples (Florence and Granada) separated. The variable “sex”, according to the origin of the participants, produced differences in the means and data dispersion, with a small effect size (Table 4). In Florence, the women showed themselves to be more convinced that ICT offered many opportunities for attending to diversity and that ICT increased motivation for learning (dimension 1—opinion), though requiring greater effort, dedication and training. They also stood out in knowing how to choose specific ICT according to student needs (dimension 2—requirements and possibilities) and in knowing how to search for and design specific activities, despite the limitations these entailed for students with FD (dimension 3—training).

**Table 4:** Significant differences in the opinion, training and requirements regarding ICT as a function of sex

City	Item	Men		Women		*t*	*d*
		*M*	SD	*M*	SD		
Florence	7	3.50	0.83	3.86	0.63	− 3.32*	0.49
	9	3.76	1.03	4.11	0.72	− 2.73*	0.40
	12	2.89	0.95	3.28	1.01	− 2.28*	0.40
	13	4.00	0.96	4.27	0.70	− 2.16*	0.32
	15	3.68	0.93	4.03	0.83	− 2.44*	0.40
	16	3.24	0.79	3.53	0.74	− 2.35*	0.38
	17	3.13	0. 91	3.53	0.75	− 3.11*	0.48
	18	3.34	0.88	3.65	0.77	− 2.33*	0.37
	19	3.13	0.70	3.43	0.85	− 2.11*	0.39
Granada	5	3.60	0.91	3.86	0.87	− 2.17*	0.29
	10	4.64	0.57	4.38	0.72	2.73*	0.40
	13	4.52	0.61	4.30	0.80	2.15*	0.31
	14	4.67	0.64	4.47	0.70	2.13*	0.30
	20	3.64	0.90	3.32	1.01	− 2.38*	0.33

*M* = Mean; SD = Standard deviation; *t* = Student’s *t*, *d* = Cohen’s *d*

*p < .05

In contrast, in Granada, it was the men who had higher scores in the items concerning the possibilities offered by ICT (Dimension 1—opinion), the training and investment they required (Dimension 2—requirements and possibilities), and the preparedness for helping students with using ICT (Dimension 3—training). The female teachers from Granada only scored highest in item 5, on the ease of use of ICT with students with FD.

The variable “years of experience with FD” revealed differences between the educators from the two countries. The participants from Florence (Table 5) without experience were more in agreement than the rest of the professionals, with a small effect size, in opining that ICT required assistance (2), gave greater flexibility (3), made it possible to meet student needs (4), were easy to use (5), and promoted inclusion (6). Furthermore, with a medium effect size, they also indicated that ICT improved a teacher’s competences (1) and made it possible to pay more attention to diversity (15). The teachers with more than 21 years of experience were those who most stated that ICT improved performance and efficacy (8), making it possible to achieve objectives in a more flexible way (11), and requiring specific training (13). The participants with fewer than 5 years of experience were more in agreement that ICT made it easier to design activities (21) and required greater dedication and effort (12). The participants from Granada (Table 6) with between 6 and 10 years of experience had sufficient knowledge to know which ICT to choose according to student needs (16) and the limitations students could have in their use (17). Compared to the teachers without experience, those with more than 21 years indicated having greater knowledge on the many opportunities of ICT for students with FD (7) and the different internet sites where one can find specific resources (18). The participants with fewer than 5 years’ experience concurred more that ICT facilitated access to information (10).

**Table 5:** Significant differences in the opinion, training and requirements regarding ICT as a function of the experience of the teachers from Florence

Item	No years		≤ 5 years		6–10 years		11–20 years		≥ 21 years		*F*	*η*^*2*^
	*M*	SD	*M*	SD	*M*	SD	*M*	SD	*M*	SD		
1	4.25	0.61	4.14	0.84	3.68	0.93	3.62	0.98	4.09	0.83	9.05*	0.07
2	4.29	0.81	4.00	0.75	3.80	0.76	3.93	0.75	4.18	0.41	3.61*	0.03
3	4.54	0.51	4.36	0.72	4.11	0.77	4.07	0.70	4.09	0.83	4.81*	0.04
4	4.33	0.34	4.23	0.73	3.95	0.76	3.86	0.69	4.18	0.75	5.08*	0.04
5	4.17	0.70	3.99	0.81	3.78	0.89	3.79	0.73	3.64	0.92	2.69*	0.02
6	4.46	0.72	4.18	0.76	3.95	0.87	4.00	1.00	4.27	0.79	3.55*	0.03
8	4.04	0.62	3.66	0.83	3.43	0.87	3.31	0.81	4.09	0.83	5.72*	0.05
11	4.25	0.61	4.17	0.68	3.86	0.83	4.00	0.93	4.27	0.79	5.36*	0.04
12	3.62	1.09	3.33	1.02	3.06	0.99	3.41	0.91	3.64	0.51	3.56*	0.03
13	4.54	0.51	4.25	0.74	4.19	0.74	4.07	0.70	4.82	0.41	3.45*	0.03
15	4.46	0.51	4.15	0.79	3.82	0.91	3.83	0.71	3.55	0.69	7.22*	0.06
21	3.92	0.88	4.08	0.70	3.82	0.80	3.76	0.99	3.82	0.98	3.32*	0.03

*M* = Mean; SD = Standard deviation; *F* = ANOVA, *η*^***2***^ = eta squared

*p < .05

**Table 6:** Significant differences in the opinion, training and requirements regarding ICT as a function of the experience of the teachers from Granada

Item	No years		≤ 5 years		6–10 years		11–20 years		≥ 21 years		*F*	*η*^2^
	*M*	SD	*M*	SD	*M*	SD	*M*	SD	*M*	SD		
7	3.40	1.01	3.71	0.95	3.89	0.79	3.92	0.64	4.05	0.80	2.99*	0.04
10	4.08	0.95	4.53	0.65	4.47	0.55	4.41	0.67	4.29	0.84	2.93*	0.4
16	3.08	1.19	3.45	0.97	3.68	0.94	3.67	0.81	3.64	0.85	2.48*	0.03
17	3.12	1.27	3.58	0.95	3.81	0.88	3.77	0.72	3.79	0.90	3.01*	0.04
18	3.36	1.08	3.77	0.99	4.06	0.87	4.03	0.71	4.07	0.78	3.97*	0.05
21	3.60	1.23	3.99	0.85	4.06	0.73	4.00	0.80	3.79	0.75	3.45*	0.04

*M* = Mean; SD = Standard deviation; *F* = ANOVA, *η*^***2***^ = eta squared

*p < .05

## Discussion and conclusions

The fact that educators know the different possibilities that ICT offers in terms of their own development and that of their students is not only important but means that competence in this area is essential [55] for attending to all students. This study has arisen precisely because we consider that, given today’s society, ICT training for teachers is indispensable.

Overall, we can state that the opinion the participants held on ICT for attending to diversity was favorable. The general tendency made it clear that they agreed with the idea that ICT improved their professional teaching competences, which concurs with Amador et al. [10], Fernández-Batanero et al. [4] and Martínez-Abad et al. [11]. They stated that the different digital options made it possible to attend to diversity, adapt to the students [30] and promote educational inclusion [12] by providing flexibility to the teaching–learning process.

Although authors such as Fernández-Batanero et al. [4] note that ICTs require specialized effort, the participants in this study did not fully agree with this statement. However, they did assert that their use requires specific training, greater material means and assistance.

Regarding the training and experience in ICT, the tendency was not so positive. The responses were centered on the middle option. They revealed a lack of competence for designing activities with general educational software, helping students with using ICT, an ignorance of search websites for creating teaching and digital resources, and a lack of knowledge about the limitations that students with FD might find when using these technological tools. These results, as with those of Cabero-Almenara et al. [13] and Fernández-Batanero et al. [4], indicate that the digital training of teachers is meagre and limited.

The educators agreed that ICT presented benefits in terms of looking after diversity, yet they did not completely agree that digital tools improved performance and efficacy. However, they did concur in that they increased motivation for learning [30], enabling access to information, and therefore to the educational world [4]. Given the attraction that ICTs hold [2], the participants supported the idea that with them it is possible to attain objectives in a more flexible way and achieve proposed educational aims [7].

The availability of different ICTs in the schools where they worked enabled the teachers and different educators to carry out tasks and activities based on digital tools for attending to diversity. The participants mainly used the computer, tablet and interactive whiteboard, coinciding with the comparison of Italy and Spain carried out by Messina et al. [32]. These devices are the most common in classrooms, thus justifying their use as such. As has been observed, the interactive whiteboard was not the most used tool by the participants, in contrast to the findings of Ardiç [39].

The comparison between cities produced a higher appreciation and opinion of ICT with students with FD by the educators from Granada. The generally favorable predisposition of the participants from Spain toward ICT placed their means above those of the Florentines as regards their digital training and knowledge of the functionalities these tools offer in the sphere of attending to diversity.

Sex proved to be a determinant. The female Florentine educators highlighted the dedication and training that ICTs in the classroom require, and their impact on the quality of teaching students with FD. Furthermore, they showed a higher digital competence, in contrast to the results obtained by Randazzo and Oteri [31], who did not discern any differences according to sex in their study on Italian teachers. The tendency in Granada was different: the men felt more prepared to help and support their students.

Experience with diverse students also proved decisive. The Florentine participants with more experience were shown to be more in agreement over the statement that ICTs require specific training. These results suggest that the most experienced educators understand the relevance of ICT for their own performance and the importance of their training, which concurs with the conclusions reached by Randazzo and Oteri [31]. Those educators lacking experience with students with FD were differentiated from the rest in considering that the different technologies available in the classroom helped such students, promoting more flexible teaching processes, meeting their needs and favoring their inclusion, even though ICT required assistance to use.

The participants from Granada with 6 to 10 years of experience were shown to be more competent in choosing specific ICTs for students with FD, being aware of the limitations that these students might find in using them. These findings are in agreement with Nikolopoulou et al. [35], whereby teachers with less experience are more willing to use educational technology. The veteran teachers were aware of the possibilities offered by ICT [4], and knew how to find websites with specific materials to utilize.

ICTs, as we have been asserting, are an innovative option and much in vogue, both in the educational and social sphere. It is practical and essential that in the subject of diversity there is training with different technologies and education regarding their use, given that they are present in the daily life of everyone. The task of ensuring that educators of schools and associations that work with people with FD acquire the necessary knowledge for applying methodologies based on ICT is arduous but worthwhile for the sake of the comprehensive development of people with different capabilities.

Moreover, ICT does not only offer benefits to students. The tendency of the results of this study has shown that their use in the classroom fosters the professional and digital competence of the educator and enhances their critical thinking, as Yilmaz demonstrates (2021). We did not see a lack of awareness regarding the potential of educational technology, as shown in Fernández-Batanero et al. [4], thus meaning that our results favor the idea that digital tools make a more personalized form of teaching possible [34]. However, such tools necessarily require more resources and the updating of specialized training so that they can be implemented and incorporated fully in the different contexts of education with people with FD.

Among the limitations of this study, we should point out that the only method of information gathering used was self-reporting. Future studies could be complemented with a more comprehensive and qualitative approach, giving a voice to the participants and ICT users themselves, and they could also implement programs to quantitatively measure the effectiveness of using ICT with students with FD. Another limitation concerns the type of sampling conducted, meaning that the results should be treated with caution. The number of participants from the Italian and Spanish contexts should be expanded and include others from elsewhere, in order to gain a worldwide panorama of the use of educational ICT. Other interesting considerations to explore would be to differentiate between educational stages, types of schools, socioeconomic levels, rural and urban areas, availability of resources and internet, and other independent variables.

## Data Availability

The datasets generated during and/or analyzed during the current study are available from the corresponding author on reasonable request.
